# Prevalence of *CCR5delta32* in Northeastern Iran

**DOI:** 10.1186/s12881-019-0913-9

**Published:** 2019-11-15

**Authors:** Amir Tajbakhsh, Mostafa Fazeli, Mehdi Rezaee, Faezeh Ghasemi, Mastoureh Momen Heravi, Aida Gholoobi, Zahra Meshkat

**Affiliations:** 1 Department of Modern Sciences & Technologies, Faculty of Medicine, Mashhad University of Medical Sciences, Mashhad, Iran; 2 Pharmaceutical Sciences Research Center, Shiraz University of Medical Sciences, Shiraz, Iran; 3 Student Research Committee, Faculty of Medicine, Mashhad University of Medical Sciences, Mashhad, Iran; 4 Department of Medical Biotechnology, School of Medicine, Mashhad University of Medical Sciences, Mashhad, Iran; 5 Blood Transfusion Research Center, High Institute for Research and Education in Transfusion Medicine, Tehran, Iran; 6 Antimicrobial Resistance Research Center, Mashhad University of Medical Sciences, P.O Box: 9196773117, Mashhad, IR Iran

**Keywords:** HIV-1, CC chemokine receptor type 5 (CCR5), Epidemiology, Geographic spread, Rare alleles

## Abstract

**Background:**

A 32-base pair deletion (*∆32*) in the open reading frame (ORF) of C-C motif chemokine receptor 5 (CCR5) seems to be a protective variant against immune system diseases, especially human immunodeficiency virus type 1 (HIV-1). We aimed to assess the frequency of *CCR5∆32* in the healthy Iranian population.

**Methods:**

In this study, 400 normal samples from Khorasan, northeastern Iran, were randomly selected. The frequency of *CCR5∆32* carriers was investigated using PCR analysis. Allele prevalence and the fit to the Hardy-Weinberg equilibrium were analyzed.

**Results:**

The prevalence of *CCR5∆32* in the northeastern population of Iran was 0.016. Four hundred samples were studied, among which one with *CCR5*^*∆32/∆32*^ and 11 with *CCR5*^*Wild/∆32*^ genotype were detected.

**Conclusion:**

This study was the first investigation for an assessment of the prevalence of *CCR5∆32* in northeastern Iran. The low prevalence of *CCR5∆32* allele in the Iranian population may result in the increased susceptibility to HIV-1. In addition, this prevalence is the same as that of reported in East Asia, while is lower than that in the Europeans.

## Background

Genetic mutations play an important role in the susceptibility and the progression of various human diseases in populations [1]. Chemokines are low-molecular-weight cytokines, which bring leukocytes to the sites of inflammation, infection, or injury. The interaction of chemokines with their receptors can locally control the progression, recruitment, and induction of lymphocytes. Therefore, the chemokine receptors are utmost important in the immune response against pathogens and inflammatory responses [2]. C-C chemokine receptor 5 (CCR5) is a seven passed transmembrane G-protein-coupled receptor of which variations could elucidate the reason for high susceptibility or the resistance of individuals to a specific infectious disease [2, 3]. The CCR5 is a co-receptor involved in the human immunodeficiency virus (HIV) entry into the target cells in the initial phases of infection. The HIV type 1 (HIV-1) attaches to CCR5 on monocytes and macrophages through the infection process [2].

**Fig. 1:**
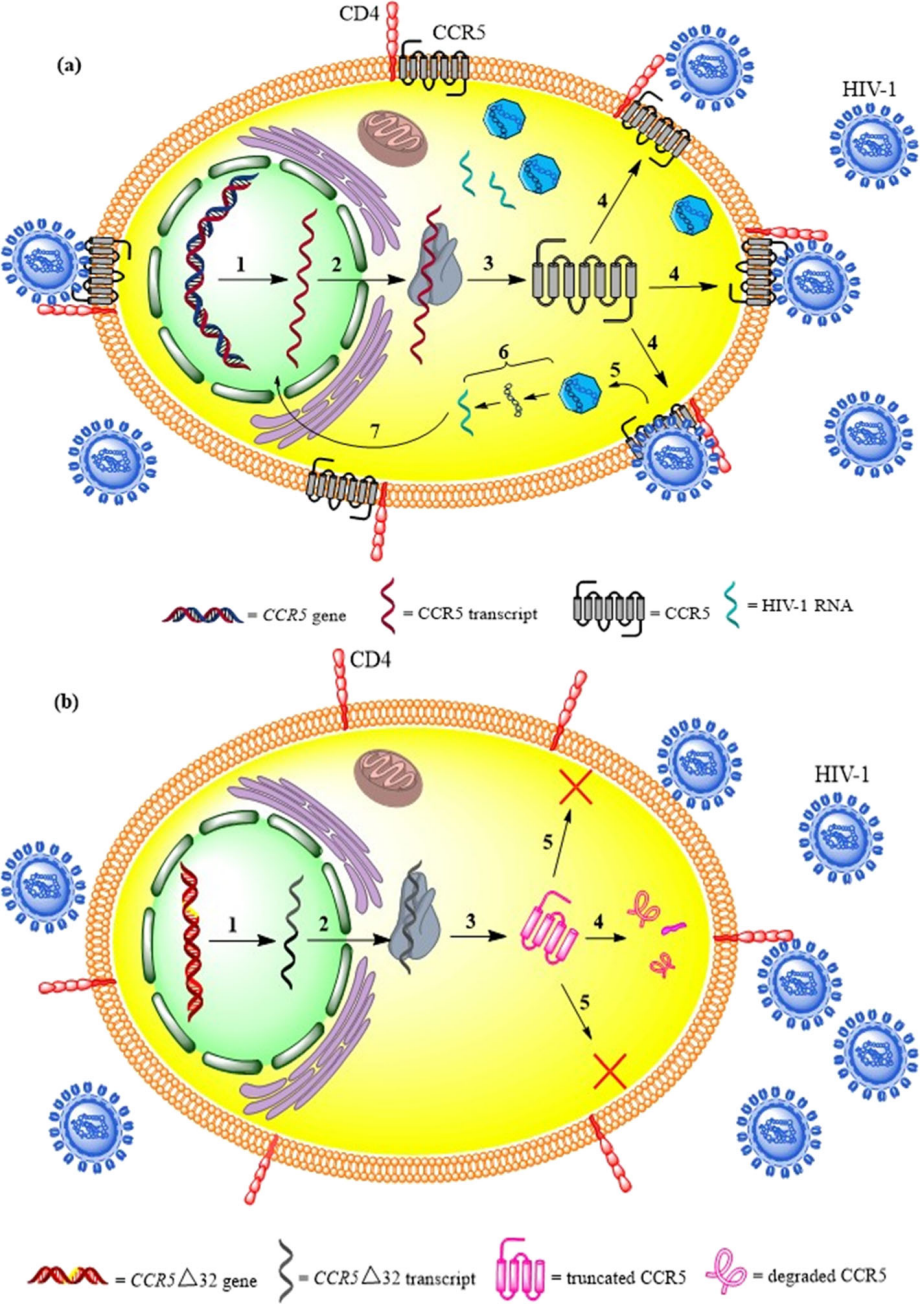
Scheme 1 shows the role of *CCR5Δ32* in protection against HIV-1 infection; (**a**) The normal cell with wild type CCR5 gene: 1. transcription, 2. mRNA transfer to the cytosol, 3. translation, 4. conformation and transferring to the cell membrane, 5. HIV-1 attachment and entry, 6. production of HIV-1 RNA, 7. transferring HIV-1 RNA to the nucleus; (**b**) A cell with *CCR5Δ32* gene: 1. transcription, 2. mRNA transfer to cytosol, 3. translation, 4. wrong conformation and degradation, 5. the absence of CCR5 on the cell surface and naught HIV-1 entry through CCR5

The genetic variations of chemokine and chemokine receptor genes are paramount in their structures and functions. A 32-nucleotide deletion in the exon of *CCR5* gene (*CCR5∆32*) has a considerable influence on the attachment capability of HIV-1 to CCR5, leading to a defective phenotype of the receptor [3]. The *CCR5∆32* causes a frame shift and premature stop codon which results in dysfunction of CCR5 [4]. Meanwhile, the *CCR5∆32* gene produces a truncated CCR5, which cannot be transported to the cell membrane [5] (Fig. 1). The absence of CCR5 on the cell surface prevents the cellular entry of CCR5-tropic (R5-tropic) HIV-1 strains into the cells [6]. The individuals with homozygote genotype for *CCR5Δ32* (*CCR5*^*∆32/∆32*^) are protected against repeated exposure to HIV-1 infection. The CCR5^∆32/∆32^ causes resistance to HIV infection, while the *CCR5Δ32* heterozygote genotype (*CCR5*^*Wild/∆32*^) considerably hinders the onset of AIDS but is not quite protected against it [7]. The *CCR5*^*Wild/∆32*^ genotype is significantly associated with slower HIV-1 disease progression and better response to treatment compared to the wild-type genotype [8]. The *CCR5*^*Wild/∆32*^ T-cells express lower CCR5 than normal T-cells, resulting in lower HIV infection [9, 10]. In addition, a study showed that the *CCR5*^*Wild/∆32*^ genotype caused 2–4 years slower development of AIDS following HIV-1 infection compared to the *CCR5*^*Wild/Wild*^ genotype [5]. Moreover, it is also shown that the HIV-1 viral load was 6- to 8-fold lower in *CCR5*^*Wild/∆32*^ compared to *CCR5*^*Wild/Wild*^ [5, 11]. Therefore, the CCR5 is an excellent target to develop novel therapeutics for HIV treatment. As there are different frequencies of *CCR5∆32* worldwide, we aim to assess the prevalence of the *CCR5Δ32* in northeastern Iran (Khorasan Province) for the first time and specify the origin of these genotypes in Iran compared to other countries.

## Methods

### Study population

In this line, we received 400 blood samples of HIV-negative healthy subjects of the Mashhad cohort study (Grant number: 85134; Mashhad University of Medical Sciences, Khorasan northeastern Iran) [12]. The MASHAD cohort study has begun in 2010 in the north-eastern Iran. Individuals were collected from three regions. In this line, each region was separated into nine sites centered [12]. There were 27 clusters in the Mashhad cohort study, which 15 samples of each cluster were randomly selected by the technique of stratified cluster random sampling. In this regard, these samples were almost age- and sex-matched that were included in this study [12]. It is worth mention that we selected healthy individuals without HIV infection or cardiovascular events. Thus, cardiovascular events are not a limitation of our study. For the purpose of this study, the following key data were also extracted from Mashhad cohort study [12]. The extraction of DNA from blood samples was done using Genomic DNA Extraction Kit (Genet Bio Company; Korea).

### Genotyping

The samples were genotyped by amplification of the region containing *CCR5Δ32* using PCR assay. PCR genotyping was experimented as described previously [9]. The forward and reverse primers were as follows, respectively: (5′-AGGTCTTCATTACACCTGCAGC-3′), and (5′-CTTCTCATTTCGACACCGAAGC-3′). It is noteworthy that genotypes were detected according to the final size of PCR products, of which 169 bp and 137 bp products were related to the wild type and the *CCR5∆32* genotypes, respectively. Each PCR reaction was experimented in 25 μl containing 5–10 ng of the purified DNA sample (1500 μmol), 1 unit of *Taq DNA polymerase* (CinnaGen Company; Iran), 2.5 μl PCR Buffer (10X) (300 μmol), 10 pmol/μl of the reverse primer, and 10 pmol/μl of the forward primer for detecting the *CCR5∆32*. The PCR reaction was used the Applied Biosystems PCR (Life Technologies Company; United States), under the following thermal conditions: initial denaturation for 3 min at 95 °C; 35–40 cycles at 94 °C for 30 s, 58 °C for 30 s, 72 °C for 30 s, and finally elongation at 72 °C for 7 min. In the following, PCR products (7 μl) were electrophoresed on a 2.5% agarose gel (Invitrogen Company; United States) stained with DNA Green Viewer (Pars Tous Company; Iran) using an electrophoresis analyzer (Consort BVBA Company; Germany) and SYNGENE U; GENIUS gel documentation system.

### Statistical analysis

The genotyping data were analysed via the SPSS 20.0 (IBM Inc., Chicago, IL, USA). The genotype and the allele frequencies of *CCR5D32* variant were calculated using gene count and the χ2 test. Moreover, Hardy–Weinberg Equilibrium (HWE) assumption was investigated by the Pearson χ^2^ distribution. In this study, *P*_*-values*_ of *p* ≤ 0.05 were considered to be statistically significant.

## Results

**Fig. 2:**
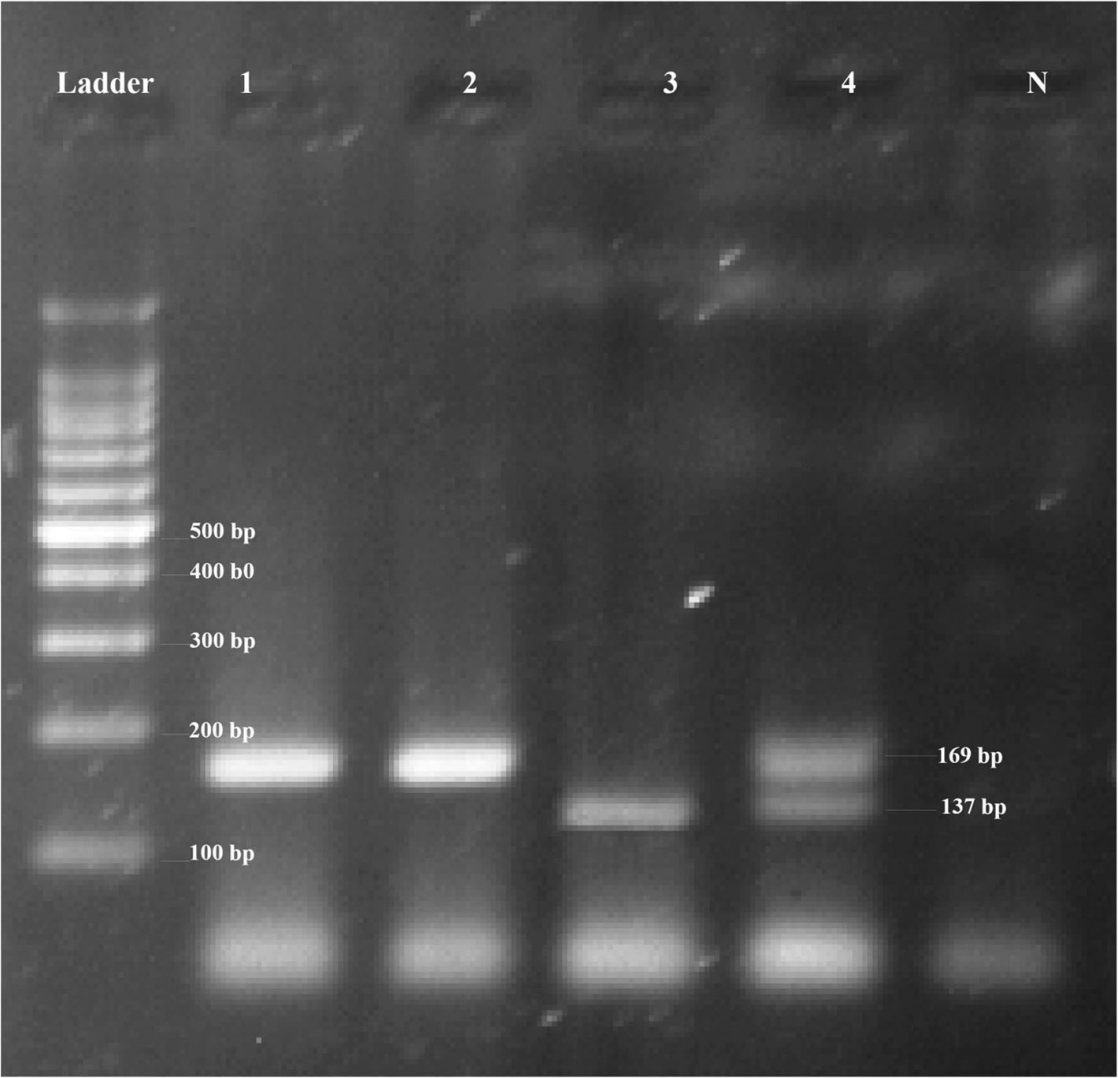
The gel electrophoresis of PCR amplified DNA with *CCR5∆32* allele. Lanes 1, and 2, wild type (*CCR5* ^*Wild/Wild*^); lane 3: mutant type (*CCR5* ^*∆32/∆32*^); lane 4: heterozygous (*CCR5* ^*Wild/∆32*^); ladder: 100 bp DNA size marker; N: negative control

The demographics of study subjects are summerized in Table 1. The 169-bp band represented the wild-type alleles and the 137-bp band represented the mutant genotype (Fig. 2). Totally, 12 mutant alleles (11 heterozygotes and one homozygote) were detected among all the samples (Table 2). The prevalence of *CCR5∆32* allele was 0.016. Although, in this study, all the samples were randomly selected based on the statically selection, data analysis was indicated that allele and genotype distribution of our samples were not in the HWE (*P-*_value_ = 0.020).

**Table 1:** The characteristics of study samples

	Wild type	Mutant^b^	Heterozygote	Total
Sex^a^	46.1 (m), 53.9 (f)	100.0 (f)	45.5 (m), 54.5 (f)	45.9 (m), 54.1 (f)
Age (yr)	46.09 ± 7.25	37.00	46.45 ± 8.12	46.1 ± 7.27
Weight (Kg)	71.92 ± 13.27	61.2	77.11 ± 11.8	72.04 ± 13.2
Height (m)	1.62 ± .09	1.51	1.64 ± .10	1.62 ± .1
BMI	27.5 ± 4.79	26.84	29.21 ± 6.29	27.5 ± 4.8
Inflammatory marker				
Hs-CRP	1.64 (3.11–1.00)	0.75	1.45 (3.40–0.87)	1.62 (3.10–1.00)
Smoking				
Non-smoker^a^	63.5	100.0	81.8	64.1
Ex-smoker^a^	10.0	0.0	18.2	9.7
Current smoker^a^	26.5	0.0	0.0	26.2
Diabetes				
Diabetic condition^a^	15.4	0.0	20.0	15.5
Biochemical parameters				
Glucose (mg/dL)	82.0 (92.0–74.5)	71.00	82.0 (96.0–74.0)	82.0 (92.0–74.5)
Cholesterol (mg/dL)	187.00 (214.00–163.00)	175.00	182.00 (233.00–153.00)	187.00 (214.00–163.00
HDL (mg/dL)	41.5 ± 9.0	39.30	41.43 ± 10.82	41.5 ± 9.0
Triglyceride (mg/dL)	116.0 (172.0–81.50)	74.00	92.00 (210.00–74.00)	115.0 (172.0–81.50)
LDL (mg/dL)	118.88 (141.6–95.75)	102.06	121.88 (160.80–84.00)	118.92 (141.6–95.75)

The results showed as mean ± SD and median (IQ3-IQ1) for normal and abnormal distribution data, respectively. ^a^ The numbers represent percentage of prevalence; ^b^ mutant group have only one subject

**Table 2:** The results of the genotyping samples

Genotype form	Genotype frequency (number (%))				Allele frequency	
	Normal homozygote CCR5 ^Wild/ Wild^	Heterozygote *CCR5* ^Wild/∆32^	Mutant homozygote *CCR5*^∆32/ ∆32^	Total	Wild	∆32
Observed	388 (97%)	11 (2.75%)	1 (0.25%)	400	0.983	0.016
Observed Frequencies	0.97	0.027	0.002	1		
Expected	387.105	12.788	0.105	400		
Expected Frequencies	0.967	0.031	0.0002	1		
X^2^=	0.002	0.250	7.573			
Overall X^2^=	7.825337719	Overall (P_*-value*_) = 0.020				

## Discussion

Chemokine receptor CCR5 plays a critical role in the entrance of HIV to the host cells and accordingly in the progression of AIDS. Hence, CCR5 is considered as a potential target for both prevention and treatment of HIV infection. Discovery of *CCR5∆32* has opened a new field in the treatment of HIV-1 infection. In this case, the investigation of allele distribution in various populations is informative [13]. The *CCR5* variations could explain why some people are more susceptible to AIDS than others [11, 14]. Few studies have focused on the genetic susceptibility of Iranian population to HIV-1. The frequency of *CCR5*∆32 allele in the normal population of North East of Iran has not been investigated. Hence, in the present study, we describe the prevalence of *CCR5∆32* among the Iranian population. Moreover, we observed a HWE deviation in this population. It could be due to some reasons, including: *CCR5∆32* has a low allelic frequency, and a mixture genetic in this population.

The discuss origin of *CCR5∆32* allele is still controversial. The *CCR5*∆32 allele is mostly considered in the Europeans population [15]. However, this allele has been observed in East and South-East Asian, African, and Indo-American populations [15]. Caucasians are widely distributed in Eurasia, the Middle East, and North Africa [16]. Moreover, the frequency of *CCR5∆32* within the Caucasians is different [15]. Furthermore, the *CCR5*∆32 allele is rarely indicated in the population of North Africa and the Middle East Arabs. The allele distribution in the Europeans has been shown to be distributed in a north-south downhill manner. Several reasons have been noted for distribution of the allele in the Europeans such as population migration, genetic admixture and outbreaks of infectious diseases [17].

Phylogenetic studies have demonstrated that the Iranians are similar to population of northern India, the Greeks and some European populations such as Italian, English, German, Finn and Lapp [16]. However, the *CCR5∆32* allele frequency in Iranian population is observed less than the Europeans [18]. Historical and phylogenetic evidence have suggested that Iranian and European populations are divided from a common ancestral population being called Indo-European [19].

According to several studies, the Vikings played a major role in the distribution of the *CCR5∆32* allele in Europe. The *CCR5*∆32 allele is graphically distributed in Europe, Eurasia, and Anatolia in coincidence with the area which the Vikings were dominant [20, 21]. It seems that Vikings are involved in introducing this mutated allele and its related disease to the countries and changed its incidence in the target populations [20, 21]. There is also a strong positive association between *CCR5*∆32 allele prevalence and the geographic and climatic factors [15]. Historical data suggests that the more combination of Eastern population of Iran with the Mongol invaders and other Eastern nations could have diluted the *CCR5∆32* allele prevalence. The high prevalence of the *CCR5∆32* allele in the northern and the north-western population of Iran can be contributed to the age of the Vikings or can be due to the less combination with attackers whose allele prevalence was less than that population. The prevalence of *CCR5*∆32 allele is decreased from North to South of Iran, similarly, the allele prevalence both in North East (according to the results of this study) and in South East of Iran was very low [22–24] (Table 3). This is while the prevalence of this allele was higher in the North and North West of Iran [24, 25]. In 2005, Gharagozloo et al. reported the prevalence rate of *CCR5∆32* to be 0.0146 among normal individuals in the South of Iran [26]. In 2014, Rahimi et al. reported that *CCR5∆32* allelic prevalence was 1.1 and 0.19% for heterozygous and homozygous respectively genotypes in populations of several provinces of Iran [18].

**Table 3:** Comparison of *CCR5∆32* allele distribution in Iranian healthy individuals

Author	Geographical position in Iran	Provinces	Allele frequency	Number of healthy individuals		HWE
Present study	Iran (North East)	Khorasan	0.0162	400		0.020
Arababadi et al.*,* [23]	Iran (South East)	Kerman	0.0033	300		0.99
Shahbazi et al.*,* [24]	Iran (North)	Golestan	0.0900	380		0002
Omrani et al.*,* [25]	Iran (North West)	Uromia	0.0105	190		0.99
Gharagozloo et al.*,* [26]	Iran (South)	Fars	0.0146	395		1.0
Rahimi et al.*,* [18]	Iran	Hormozgan	0.0057	30	530	0.003
		Gilan		20		
		East Azarbaijan		50		
		West Azarbaijan		50		
		Ghazvin		45		
		Tehran		100		
		Semnan		30		
		Kurdistan		35		
		Ghom		30		
		Isfahan		40		
		Yazd		30		
		Khorasan		40		
		Lorestan		30		
Heydarifard et al.*,* [27]	North	Gorgan	0.0150	300		0.7920
Bineshian et al.*,* [28]	Between Center and North	Tehran	0	100		–
Abdolmohammadi et al.*,* [29]	North	Golestan	0.072	455		0.004

In this case, the allele frequency in the north of Iran (Golestan Province) with different ethnicity and population is higher than another place in Iran. In this line, Shahbazi et al. and Abdolmohammadi et al. identified that allele frequency of *CCR5∆32* were 0.09 and 0.072, respectively in north of Iran [24, 29]. Since Golestan Province is already located in the southeast of Caspian Sea, it is supposed to display an higher rate of this polymorphism but due to the presence of different ethnicities living (like Turkmen), the mutant genotype is more prevalent [27]. The different result between Iranian studies may due to genetic diversity among Iranian population [30, 31]. Investigation of genetic systems has been indicated a heterogeneity among Iranian population. Mehrjoo et al.*,* in a genome-wide association study, indicated that there is a distinct genetic diversity and also heterogeneity of the population of Iranian [32]. Comparison of gene distributions with the small number of samples of Iranian population confirmed an intra-ethnic and wide overall genetic mixture in the Iranian population. The genetic diversity reflects the differences in the structure of Iranian populations [30]. Generally, Iran is an ethnically diverse population, comprising of different groups including Pars, Lur,, Kurd, Baloch, Arab, Turkmen, and Turk [30]. Moreover, the complete mtDNA sequence analysis exposed a high genetic diversity in the Iranian population [31].

Historians believe that the most Iranians are Aryan; however they have been mixed with different foreigners during the history, for example Macedonians, Arabs, Turks, and Mongols. Moreover, Iran has a key role in linking different populations in the Silk Road, between Asia and Europe [33]. Ongadi et al. in a systematic review analysis revealed that the *CCR5Δ32* allele frequency is at 93% in Caucasians and 7% is in the other populations [34]. The frequencies of this allele in some European countries were demonstrated that is moderate such as Italy (3%), Cyprus (2.8%) and Greece (2.4%), but a high frequency (9.21%) was reported in southwest Germany [35, 36]. Moreover, this frequency is about 4% in Brazilian populations [37]. It is indicated that the distribution of *CCR5-∆32* is very low in the south of Middle East and also Arabic countries. According to our study, we observed a low frequency of mutant allele in Iran that it is in almost agreement with the result observed from countries such as Saudi Arabia, and India (1%) [38, 39]. The frequency of *CCR5-∆32* allele was indicated in Turkey (3.17%), Afghanistan (3.86%), Pakistan (2.86%) [40, 41].

On the other hand, approximately 0.8% of adults are living with HIV based on the latest data from the WHO [42]. In regions and countries, the burden of the epidemic is different [42]. The prevalence of adults living with HIV is 7.0, 1.5, 0.2, 0.2, 0.4, 0.9, 1.2 and < 0.1% in Eastern and Southern Africa, Western and Central Africa, Asia and the Pacific, Western and Central Europe and North America, Latin America, Eastern Europe and Central Asia, The Caribbean, and Middle East and North Africa [42]. Moreover, the prevalence of HIV among the general population in Iran remains low [42]. In Iran, the main populations at risk of HIV infection are people who inject drugs, prisoners and sex workers [43]. The general population category consisted mainly of research on blood donors in Iran [44]. In Bagheri’s systematic review, the prevalence of HIV in the general population was 0.00% [44]. Importantly, in a study by Haghdoost et al. is indicated that a change in the prevalence of HIV infection from people who inject drugs to the general population. This shift may due to the enhancing rate of premarital and also extramarital sexual contact, particularly with female sex workers in Iran [45]. It also demonstrated that HIV/AIDS burden was not distributed equally among different Iranian provinces, and in some provinces such as Kermanshah, Hormozgan, Lorestan, and Tehran it was more concentrated [46]. Remarkably, no case with HIV infection was detected in the general population of Mashhad [47]. Likewise, the prevalence of infection with HIV in the Iranian population of thalassemia and hemophilia and blood donors was low [48].

Beside genetic modifications, other critical immune factors that may prevent HIV-1 infection are certain chemokines and also their receptors. In this case, the CCR5 binding chemokines include CCL3, CCL4, and also CCL5 have a function as the main natural factors that act as a suppressor of HIV-1 [49]. CCL3L1 up-regulation results in the down-regulation of CCR5 and following the internalization of receptor [50]. The trans-activating function of Tax protein 2 is attributed to an increased secretion of CCL3L1 [49]. During human T-cell lymphotropic virus type 1 (HTLV)-1 and HTLV-2 infections with CCLs and CCRs, Tax1 and Tax2 may increase innate immunity in the extracellular environment, which may play a major role in regulating innate immunity during co-infection with HIV/ HTLV and inhibiting CCR5/HIV-1 [51]. The CCL3L1 down-regulates CCR5 for the entry of HIV-1, resulting in a long-term non-development status in co-infected patients with the high infection of HTLV-1 and 2 [52]. The most affected HTLV-1 cell is CD4^+^ T cell [53]. HTLV-1 and -2 are main co-pathogens among HIV-infected patients [54]. In this line, HTLV-2 and HTLV-1 infections can trigger the participation of innate HIV-1 immunity by modifying CCR5/HIV-1 binding and HIV-1 development in patients with co-infection [54]. In this regard, CCR5 down-regulation was reported for lymphocytes from HIV-1/HTLV-2 co-infected individuals [54]. High levels of co-infection with HIV-1/HTLV appear in HTLV-1-endemic regions, where HTLV-2 is transmitted by sharing the needle. In European and United States studies, individuals with HIV-1 and HTLV-2 co-infections were found to result in altered clinical outcomes, and also delayed development of AIDS [55, 56]. In contrast, there are several reports were indicated that co-infection with HTLV-1/HIV-1 is associated with faster AIDS clinical progression and shorter survival time and also have more risk to progress myelopathies as well as neurological disease [54, 55, 57, 58]. HTLV-I is widespread in a variety of geographic regions, including Japan, the Caribbean, South America, Africa and Northeastern Iran [59–61]. HTLV-I is endemic in five Iranian provinces such as Khorasan Razavi (Mashhad), Northern Khorasan, Alborz, Eastern Azarbayejan, and Golestan [61–63]. However, there is no report of co-infection HTLV-1 and HTLV-2 infection with HIV in Mashhad in the general population [47, 64]. Rahimi et al. indicated that HTLV-I/HIV co-infection may stimulate HIV replication and also could decrease the HTLV-I viral load, in infected cells in non intraveneous drug users in Mashhad [62].

In addition to general and main key population, there is a low risk of HIV-1 infection for HIV-1 laboratory workers as we all health care workers that are prolonged laboratory exposure to concentrated HIV-1 and also exposure to experiencing needle stick injuries in clinical and laboratory research [65, 66]. It is suggested that strict biosafety level 3 containment as well as practices are needed for work with HIV-1, particularly concentrated HIV-1 [65]. Although the frequency of HIV in the general population is low, it is higher in the high-risky populations such as persons who inject drugs, prisoners, and sex-workers. In this regard, the laboratories dealing with the latter group of populations are exposed to danger. Moreover, the lack of biosafety level 3 containment is another risk factor. Thus, the laboratories and the staffs researching on HIV are at risk, and most of the staffs are not interested to work in such a risky environment due to unwanted incidents. So, it is reasonable to employ the staffs, carrying the mutation (*CCR5∆32*), for working in such risky environments and blood samples.

In addition, the bioinformatics analysis indicated that mutated proteins lost three alpha helices, as the results of this changes degraded in the cells. Nevertheless, modeling indicated that the truncated protein also have the required domains for virus attachment and these domains did not show major conformational alterations with the wild type ones, so we can conclude that displaying the truncated protein on the cell surface may be a possible way of virus entry [5]. Thus, defective protein destruction in the cell and the absence of its surface display can be the main reason for *CCR5∆32* variants resistance. These findings can suggest strategies for combating against HIV infections based on the prevention of expression or surface display of CCR5 [67–72]. RNAi technology can be used to prevent of CCR5 expression or the masking of CCR5 on the cell surface which may be considered as research area to the prevention of HIV infection. Nowadays, various therapies are used to treat HIV-1 by targeting CCR5 receptors like CCR5 inhibitors. The CCR5 inhibitors include various agents such as maraviroc (MVC) (FDA approved), CMPD167, vicriviroc (VVC), aplaviroc (AVC), VCH-286, TAK-779, G-protein-coupled CCR5 receptors, zinc finger nucleases, and cell-specific RNA aptamer [73–78]. These inhibitors change the shape of CCR5 and inhibit HIV-1 entry to target cells by preventing the binding of viral protein gp120 to the CCR5 [70]. Moreover, it is indicated that introduction of the *CCR5*^*∆32/∆32*^ in induced pluripotent stem cells by the combination of clustered regularly interspaced short palindromic repeats (CRISPR)-associated protein nuclease (Cas)-9 system and a PiggyBac transposon lead to a resistance to the infection of HIV/AIDS. Moreover, a resistance was established by engineered induced pluripotent stem cells-derived phagocytic cells (monocytes and the macrophages) [67]. Based on the previous studies, a tropism-dependent resistance against HIV/AIDS infection is described in disrupts human CCR5 T-cells. In this line, in CCR5-modified cells, targeting of CCR5 via CRISPR-Cas9 technology, reduced tropic-dependent resistance against HIV-1/AIDS and has non-cytotoxic effect on the viability of cells [68, 71]. Furthermore, CRISPR-Cas9 system packaged with lentiviral vectors exposed hopeful outcomes in reducing HIV-1 infection [69].

## Conclusions

The *CCR5*∆32 allele plays a main role in the resistance to HIV-1 infection, as a natural selection allele, and also its distribution is used for geodetic survey data. Aside from its importance in the geographic distribution, the use of this mutation has brought new hope for eradicating HIV infection. Novel therapies have led to significant progress in the treatment of HIV-1 infection, whereas some side effects such as drug-drug interactions, substantial toxicity, difficulties in adherence, and increased cost remain. Therefore, with the knowledge of individuals’ genetic variations, the most efficient treatment could be chosen, which reduces drug costs and side effects with appropriate drug dosing. Furthermore, for the first time, our study revealed the low prevalence of this mutation in the normal population of North Eastern Iran (Khorasan Province) and consequently concluded that there is a HIV-1 infection. Therefore, in these areas, more attention and preventive steps should be taken to prevent HIV infection. In addition, based on the controversially results in studies, more investigation is needed to evaluate HTLV prevalence, especially HTLV-1 and its influence on the viral load of HIV as well as AIDS development in co-infected patients in endemic area such as Khorasan, Iran. Even though there are no findings of the prevalence co-infection with HTLV/HIV in Iran, which can due to low prevalence of HIV in this area, patients and healthy persons need screening for potential clinical manifestations, particularly neurological diseases. To our knowledge and based on the results of previous studies, we could not find any association between prevalence of HIV and HTLV-1 infection and *CCR5∆32* in Iran. The complete and accurate information according to the prevalence of HIV can help health authorities to design more successful plans in the general population. Since the prevalence of HIV in this area remains low, the implementation of health policies, public awareness, free HIV counseling and testing services appear to have led to this low prevalence. Conclusively, these findings provide a new understanding for scientists to define future research in the field of immunobiology of HIV-1 in Iranian population.

## Data Availability

Data sharing is not applicable to this article as no datasets were generated or analysed during the current study. However, all samples and data are collected from the Mashhad cohort study (Grant number: 85134; Mashhad University of Medical Sciences, Khorasan northeastern Iran (for more information see ref.: [12].

## Abbreviations

AVC: Aplaviroc
CCR5: C-C chemokine receptor 5
*CCR5∆32*: A 32-nucleotide deletion in the exon of CCR5 gene
*CCR5*^*∆32/∆32*^: Heterozygote genotype for *CCR5Δ32*
CRISPR-Cas9: Clustered regularly interspaced short palindromic repeats-associated protein nuclease-9
HIV: Human immunodeficiency virus
HIV-1: HIV type 1
HTLV: Human T-cell lymphotropic virus
MVC: Maraviroc
ORF: Open reading frame
R5-tropic: CCR5-tropic
VVC: Vicriviroc
